# Evaluating local plant species for effective fall armyworm management strategies in Taiwan

**DOI:** 10.1186/s40529-024-00424-0

**Published:** 2024-07-12

**Authors:** Yuan-Ching Tsai, Pei-Qi Luo, Chang-Lin Sung, Yi Li, Fang-Yu Hu, Chih-Lu Wang, Yi-Ning Chen, Ju-Hsin Hsu, Chien-En Liao, Shyh-Rong Chang, Wen-Po Chuang

**Affiliations:** 1 Department of Agronomy, National Chiayi University, Chiayi City, Taiwan; 2 Department of Agronomy, National Taiwan University, Taipei, 10617 Taiwan; 3 Division of Forage Crops, Livestock Research Institute, Ministry of Agriculture, Tainan City, Taiwan

**Keywords:** Push-pull farming system, *Spodoptera frugiperda*, *Pennisetum purpureum*, *Desmodium*, *Brachiaria brizantha*

## Abstract

**Background:**

The emergence of *Spodoptera frugiperda* (fall armyworm; FAW) in the world has raised concerns regarding its impact on crop production, particularly on corn and sorghum. While chemical control and *Bt* crops have been effective in managing FAW damage, the development of pesticide-resistant and *Bt*-resistant strains necessitates alternative control methods. The push-pull farming system has gained attention, but direct utilization of African plant species in Taiwan faces challenges due to invasive potential and climatic disparities. Therefore, identifying and evaluating suitable local plant species, such as Napier grass (*Pennisetum purpureum*), *Desmodium* species, and signal grass (*Brachiaria brizantha*), is crucial for implementing effective FAW management strategies in Taiwan.

**Results:**

In screening fifty Napier grass germplasms, all demonstrated an antibiotic effect, reducing leaf consumption compared to corn. Notably, thirty-five germplasms exhibited robust antibiotic traits, decreasing FAW consumption and increasing mortality rates. Three Napier grass germplasms also attracted more female moths for oviposition. Further evaluation of selected Napier grass germplasms and signal grass demonstrated efficacy in reducing FAW larval weight and survival duration. Additionally, *Desmodium* species, particularly *D. uncinatum*, showed promising toxicity against FAW larvae.

**Conclusion:**

Our findings support the effectiveness of selected Napier grass germplasms and signal grass as pull plants, and highlight the potential of *D. uncinatum* as a push plant in FAW management strategies in Taiwan.

## Background

The fall armyworm (FAW), scientifically known as *Spodoptera frugiperda* (J. E. Smith), is a highly destructive pest indigenous to the Americas. It has garnered significant attention due to its severe impact on crop production and resulting economic losses (Murúa et al. 2006; Wyckhuys and O’Neil 2006). The global concern escalated when FAW was first identified in Nigeria and São Tomé and neighboring African countries in early 2016 (Goergen et al. 2016). Owing to its remarkable long-distance migration capabilities, FAW rapidly spread throughout sub-Saharan Africa (Nagoshi et al. 2018), and subsequently invaded several countries in Southeast Asia, including India (Kalleshwaraswamy et al. 2018), Myanmar (Yee et al. 2019), Pakistan (Gilal et al. 2020), Indonesia (Sartiami et al. 2020), and Thailand (IPPC 2018). In January 2019, FAW was detected in Yunnan, China (Center 2019), followed by its emergence in Taiwan in June 2019. Recently, FAW has been reported in most countries of Asia and the Pacific.

FAW exhibits a broad host range, being reported in over 350 plant species (Montezano et al. 2018). Among these hosts, several major crop species, particularly corn and sorghum, suffer significant yield losses. Estimates suggest FAW could lead to yield losses of 40–45% in Ghana and Zambia (Day et al. 2017), and up to 17.7 million tons of corn yield loss across 12 maize-producing African countries (Rwomushana et al. 2018).

Currently, chemical control (synthetic pesticides) represents the most effective method for managing FAW. However, the overreliance on synthetic pesticides has led to the emergence of pesticide-resistant FAW strains (Gutiérrez-Moreno et al. 2019). Furthermore, populations of FAW in China have demonstrated resistance to pyrethroids and organophosphates (Zhang et al. 2020). Besides, transgenic crops, such as *Bt*-corn, have shown promise in pest control. However, the instances of Cry1F-resistant FAW recorded in Puerto Rico, Brazil, and the United States (Farias et al. 2014; Li et al. 2016; Storer et al. 2010).

While *Bt* crops have proven effective in controlling FAW damage, their adoption is limited, with only 26 countries currently permitting their cultivation. Therefore, finding alternative yet effective control methods, such as the push-pull farming system, is imperative (Pickett et al. 2014). In Africa, the push-pull farming system utilizes two companion plants in maize or sorghum fields. A repellent push plant, such as *Desmodium* spp., deters pests from major crops, while a trap pull plant, like Napier grass (*Pennisetum purpureum*) or *Brachiaria* grass, attracts insect pests. Additionally, the root secretions of *Desmodium* spp. suppress the germination of parasitic plants, such as Striga (Hooper et al. 2010). Moreover, *Desmodium*, being a legume, improves soil quality by increasing organic matter content and fixing nitrogen. Importantly, both push and pull plants serve as fodder for animals.

Since its development by Khan et al. (2001), the push-pull farming system has garnered significant attention. Subsequent generations have been developed to incorporate drought-tolerant traits. For the first generation, *Pennisetum purpureum* was selected as the pull plant, whereas *D. uncinatum* was chosen as the push plant (Khan et al. 2001). Due to the need for drought-tolerance traits, two additional generations of the push-pull farming system have been developed. In the second generation, *Brachiaria brizantha* cv Mulato II was selected as the pull plant, whereas *D. intortum* was designated as the push plant (Midega et al. 2015). Transitioning to the third generation, *Brachiaria brizantha* cv Xaraes was identified as the pull plant, and *D. incanum* was selected as the push plant (Cheruiyot et al. 2021).

However, the direct use of African plant species in Taiwan is not feasible due to invasive concerns and climatic differences. Therefore, to implement the push-pull farming system in Taiwan, suitable local plant species, such as Napier grass, *Desmodium* species, and signal grass, must be identified and evaluated for their efficacy in managing FAW. Taiwan is home to seventeen *Desmodium* species, usually in wet and semi-open or open thickets, wasteland, roadside or under forest (Editoral Committee of the Flora of Taiwan 1993).

In this study, fifty Napier grass germplasms, three *Desmodium* species, and one signal grass were collected and evaluated for their potential as push and pull plants in maize management in Taiwan.

## Materials and methods

### Plant material

Fifty Napier germplasms, *D. intortum*, and signal grass were obtained from Taiwan Livestock Research Institute, Ministry of Agriculture, Taiwan. The details of these fifty Napier grass germplasms are listed on the Table 1. *Desmodium tortuosum* was collected at Chiayi County, Taiwan (23.402154 N, 120.347803 E) while *D. uncinatum* was collected at Nantou County, Taiwan (24.024095 N, 121.133667 E). The cuttings were propagated within peatmoss (C102B, KEKKILA OY, Estonia), and were further maintained in the Experimental Farm, College of Bio-resources and Agriculture, National Taiwan University. For the corn plants, White-pearl, Tainan 22 (TN22) and Tainan 24 (TN24) were used in this study. Furthermore, sorghum plants, Taichung 5 (TC5) was used in this study.

**Table 1:** List of tested napier grass germplasms

Germplasms				
10 − 3	11 − 6	11 − 7	11–13	11–20
20 − 15	A9	A146	A149	DIII-14
DIV-1	DIV-4	DIV-5	DIV-7	DIV-9
DIV-10	DIV-12	DIV-14	DIV-17	DIV-18
DIV-19	DIV-20	DIV-22	G3	G4
I2	I3	I4	I5	I6
I7	I8	N1	N2	N5
N32	N36	N38	N41	P1
TS	TS2	TS3	TS4	TS5
TS7	TS8	VG3	VG5	VP4

### Insect rearing

FAW were initially collected from a maize field in Taipei, Taiwan (25.01531 N, 121.54119 E). FAW larvae were reared by artificial diet method followed the protocol by Ku (2016). Briefly, 42 g of agar was heated with 900 ml of distilled water until dissolved. The solution was then mixed with 300 g of kidney bean powder, 120 g of yeast powder, 110 g of wheat germ, 1.2 g of L-ascorbic acid, 0.75 g of streptomycin, 3 g of sorbic acid, 3.5 g of methyl p-hydroxybenzoate, and an additional 900 ml of distilled water. The larvae were maintained under controlled conditions (28 °C with a 12/12-h light/dark photoperiod.) The FAW adults were reared with 10% honey solution. Newly hatched neonates were used for the study.

### Napier grass leaf consumption area

The undamaged leaf from fifty Napier germplasms were collected from Experimental Farm, College of Bio-resources and Agriculture, National Taiwan University. The undamaged leaf of corn plants (White pearl, Known-You Seed Co., Taiwan) was also collected. The leaf was cut as the striped size and put on the petri dish (55 mm * 15 mm, Alphas Plus Scientific Corp., Taiwan) contained 1% agar. The petri dishes with striped leaves were scanned. Each germplasms have three replicates (petri dishes). Five FAW neonates were put in the petri dish and fix with parafilm to avoid the escape. After three days, the petri dish with feeding leaves were scanned. The leaf consumption area by FAW larvae was calculated by Image J (Schindelin et al. 2012). The larval mortality was also calculated.

### Field trials of napier grass germplasms

**Fig. 1:**
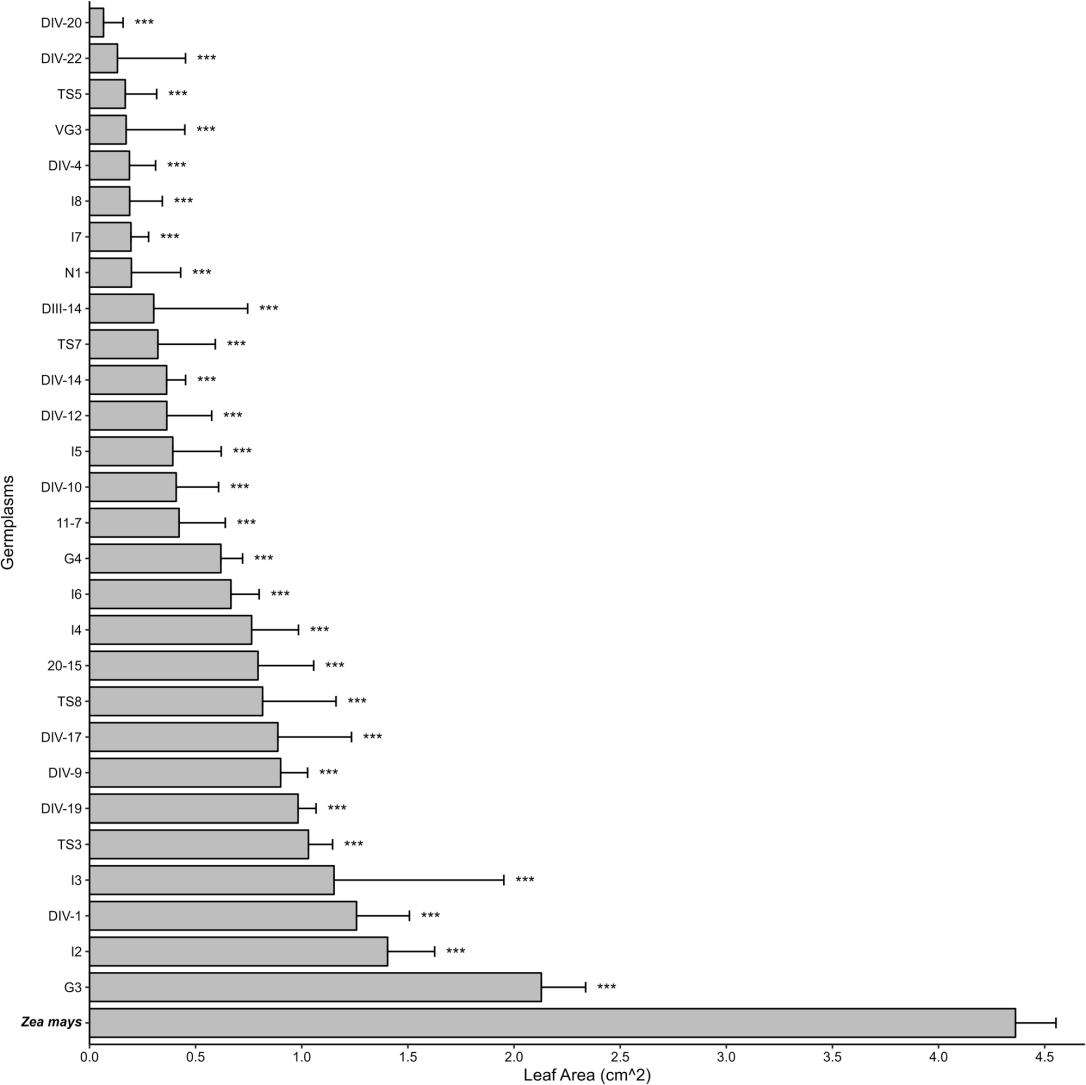
The leaf feeding area of fall armyworm fed with Napier grass and corn. Asterisks indicate differences between Napier grass germplasm and corn as *** *p* < 0.001. Error bars represent standard error (S.E.). *N* = 3

**Fig. 2:**
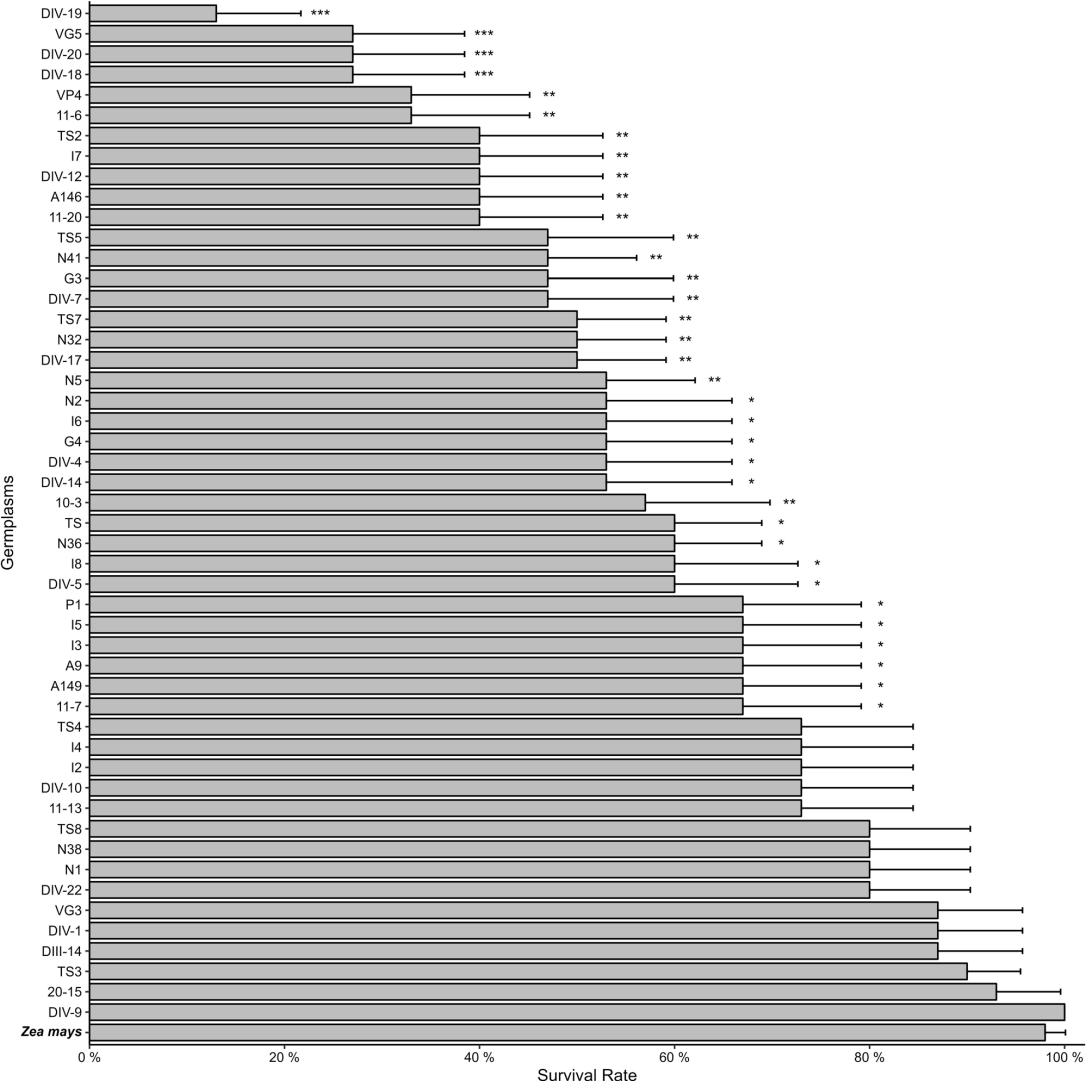
The survival rates of fall armyworm fed with Napier grass and corn. Asterisks indicate differences between Napier grass germplasm and corn as * *p* < 0.05, ** *p* < 0.01, and *** *p* < 0.001. Error bars represent standard error (S.E.). *N* = 3

Thirty-two Napier grass germplasms were selected based on Figs. 1 and 2. These thirty-two Napier grass germplasms, along with *Pennisetum sinese*, corn plants (TN24) and sorghum (TC5), were planted in the Experimental Farm at the College of Bio-resources and Agriculture, National Taiwan University, using a completely randomized design. Each replication comprised one plot containing corn, sorghum, and 11 tested *Pennisetum species*. Thus, three plots constituted one replication, and the entire experiment was replicated three times. Regular scoring was conducted to assess damage severity using a 1–5 point scale based on the criteria defined by Davis et al. (1992). Scale 1 indicates no visible damage, while Scale 2 is characterized by the presence of only pinhole lesions on whorl leaves. Scale 3 includes pinholes and small circular lesions on whorl leaves. At Scale 4, several small to mid-sized elongated lesions (1.3 to 2.5 cm in length) appear on a few whorl and furl leaves. Finally, Scale 5 features several large elongated lesions (greater than 2.5 cm in length) on a few whorl and furl leaves, and/or a few small to mid-sized uniform to irregularly shaped holes (with the basement membrane consumed) on the whorl and/or furl leaves.

The experimental field measured 20 m x 26 m. Napier grass germplasms was planted using the cutting method, where stems were retained in segments of every two clippings after removing all leaves, and direct insertion was performed, with each genotype continuously inserted into five branches. Five seeds of corn and sorghum were consecutively sown for each and scoring began on the eighteenth day. The experiment ran from June 30, 2022, to July 28, 2022, with evaluations conducted twice a week (every Monday and Thursday). The survey spanned one month, aiming to identify Napier grass germplasms with enhanced attractiveness to pests.

### FAW bioassay on napier grass germplasms and signal grass

Leaves from four Napier grass germplasms (G3, G4, DIV-7, and TS5) and signal grass (*Brachiaria brizantha*) were collected from the Experimental Farm at the College of Bio-resources and Agriculture, National Taiwan University. Additionally, leaves from corn plants (TN22) were gathered for the bioassay. All leaves had their midribs removed and were then placed into cups with 1% agar, serving as food for FAW neonates. The neonates were kept in a growth chamber (CK-68EXD, Chang Kuang, Taiwan) at 28 °C with a 12/12-hour light/dark photoperiod. The leaves were regularly replaced until the FAW larvae either died or pupated. The sample size for FAW fed on Napier grass germplasms and corn was 50, while the sample size for FAW fed on signal grass was 100.

On the eleventh day of the bioassay, the larval weight was measured. Additionally, the larval period, pupation weight, pupation rate, and the number of male and female adults were recorded. The emerged adults were transferred to circular, darkened oviposition containers, fed with cotton soaked in 10% honey, and daily collection of egg masses was conducted. Under a dissecting microscope (Stemi 508, ZEISS, Germany), egg masses were dissected with a dissecting needle to count and evaluate the oviposition capacity differences among treatment groups.

#### FAW bioassay on desmodium species

Leaves from two *Desmodium* species (*D. intortum* and *D. uncinatu*m) were collected from the Experimental Farm at the College of Bio-resources and Agriculture, National Taiwan University. Additionally, leaves from corn plants (TN24) were gathered for the bioassay. All leaves had their midribs removed and were then placed into cups with 1% agar, serving as food for FAW neonates. The neonates were kept in a growth chamber (CK-68EXD, Chang Kuang, Taiwan) at 28 °C with a 12/12-hour light/dark photoperiod. The leaves were regularly replaced until the FAW larvae either died or pupated. The sample size for *Desmodium* species and corn was 45. On the eleventh day of the bioassay, the larval weight was measured. Additionally, the larval period, pupation rate, and the number of male and female adults were recorded.

### Statistical analysis

All statistical tests were conducted using the free statistical software R (Version 4.3.1) (R C Team 2023), where the significance level was fixed at 0.05. FAW larval survival proportions in Napier grass were compared using the two-sample proportion z-test. Leaf consumption areas were analyzed using the analysis of variance (ANOVA) method. Dunnett test was further conducted to compare all treatment means. Student’s t-test was used to compare Napier grass germplasms with corn. Larval weight, larval period and pupal weight were analyzed using the ANOVA method, respectively. Based on the results of ANOVA, the Fisher’s least significance difference (LSD) test was applied to compare the means of different groups. In addition, the Bonferroni’s correction method was used to control the family-wise error rate. Pupation rate, adult emergence rate and adult gender ratio were analyzed using the two-sample z-test. Egg number per female was analyzed using the chi-square test. Larval survival curves were estimated using the Kaplan-Meier estimator and were further compared using the log-rank test.

## Results

### Evaluate the napier grass and signal grass for the pull plant

To identify an effective pull plant with traits that attract more female insects for oviposition and induce toxicity in larval development, fifty Napier grass germplasms for their resistance to FAW were evaluated. FAW neonates were introduced to these germplasms, and their consumption and survival rates were analyzed. Results indicated that all tested Napier grass germplasms displayed an antibiotic effect, resulting in reduced leaf consumption compared to the preferred host, corn (Fig. 1). It’s noteworthy that the leaf consumption area of twenty-two Napier grass germplasms could not be accurately calculated due to values close to zero. Moreover, thirty-five out of the fifty Napier grass germplasms exhibited robust antibiotic traits, showcasing both decreased FAW consumption and elevated mortality rates (Fig. 2).

**Fig. 3:**
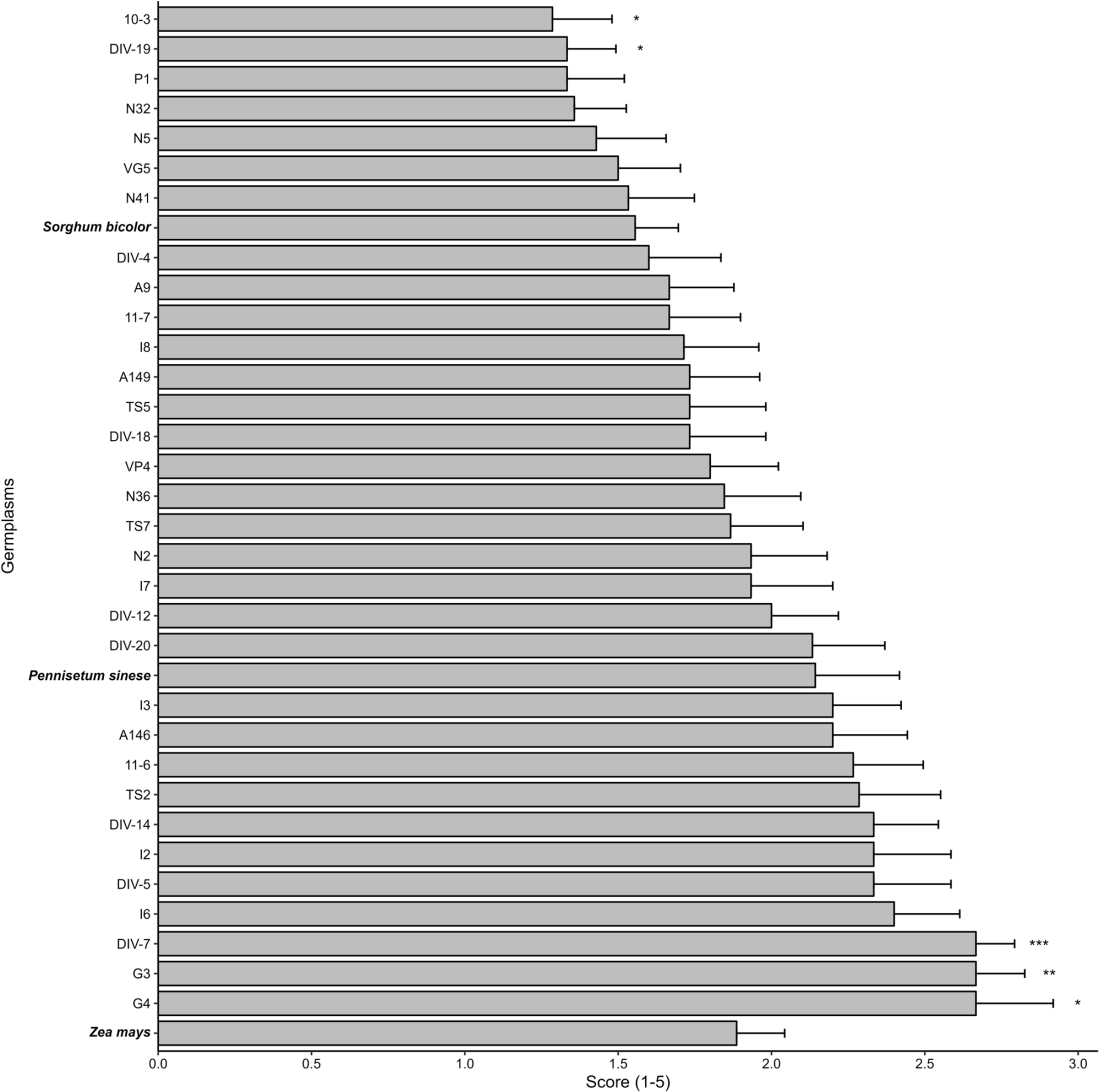
The damage scores of Napier grass germplasms, *Pennisetum sinese*, and corn in the field trial. The damage severity was based on the criteria defined by Davis et al. (1992). Asterisks indicate differences between Napier grass germplasm and corn as * *p* < 0.05, ** *p* < 0.01, and *** *p* < 0.001. *N* = 3

Since these germplasms would be utilized as pull plants in the push-pull farming system, our aim was to select Napier grass candidates with reduced FAW consumption and increased mortality rates. Additionally, we sought plants that attracted more FAW female moths for oviposition than corn. Given challenges in investigating egg numbers in the field, we employed leaf damage scores as indicators. Thirty-two Napier grass germplasms were chosen for field trials based on their favorable traits. After one month of evaluation, three germplasms (G3, G4, and DIV-7) exhibited higher damage scores than corn, signifying increased attraction to FAW female moths for oviposition (Fig. 3). However, FAW demonstrated poor performance on these germplasms in no-choice assays (Figs. 1 and 2).

**Fig. 4:**
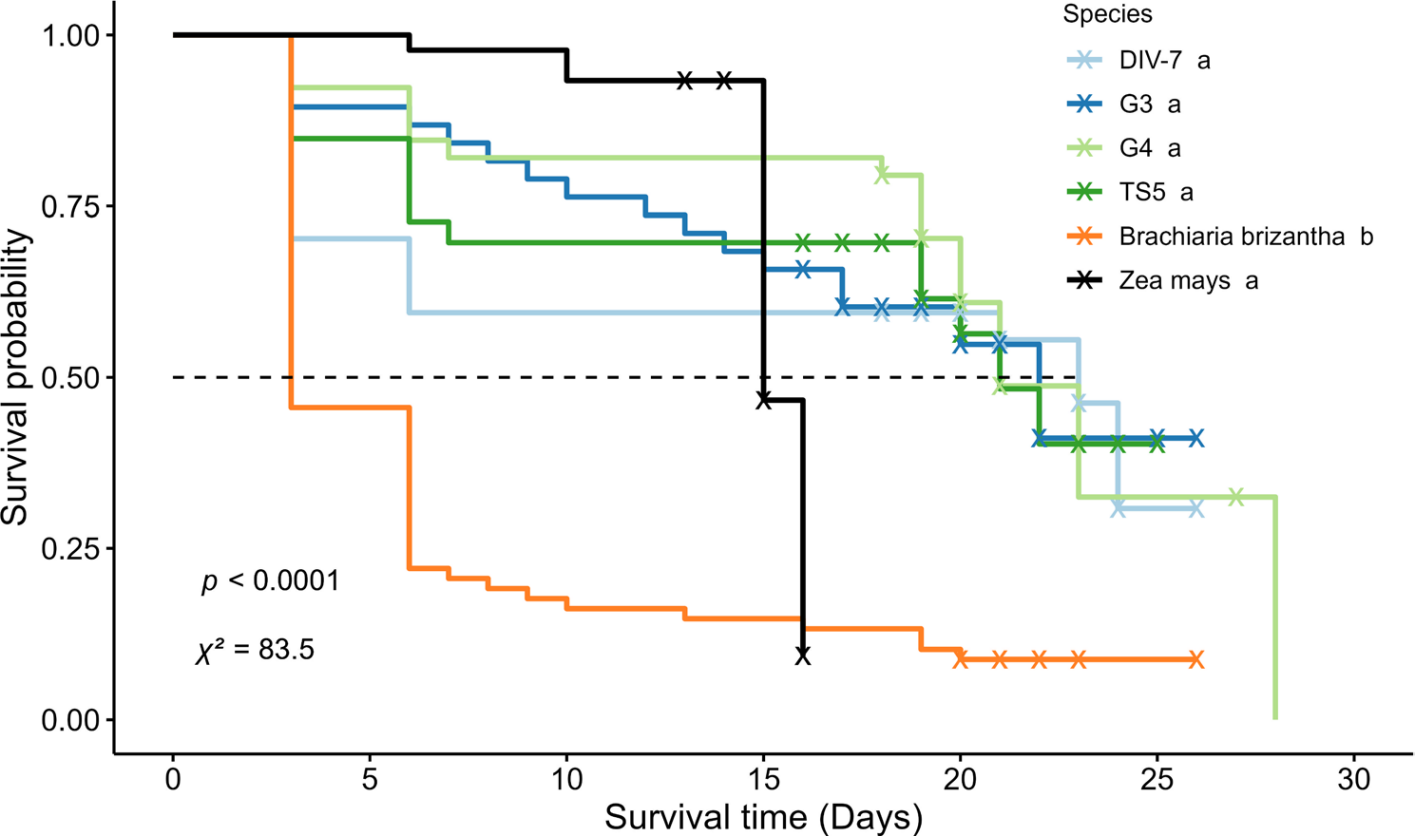
The survival probability of fall armyworm (FAW) larvae when fed on Napier grass, signal grass, and corn. The survival curves were analyzed using the Kaplan-Meier log-rank test. Labels marked with the same letter indicate no significant difference. The symbol “x” denotes censored FAW larvae

These three selected Napier grass germplasms, along with another Napier grass germplasm (TS5) and signal grass (*Brachiaria brizantha*), underwent further evaluation for their antibiotic effects on FAW development. Signal grass serves as an alternative pull plant. After 11 feeding days, FAW larvae fed on all four Napier grass germplasms and signal grass exhibited significantly reduced larval weight compared to those fed on corn plants (Table 2). However, no significant differences were observed among the test Napier grass germplasms and signal grass. Additionally, FAW larvae fed on these plants required more time to complete all larval stages than those fed on corn plants (Table 2). Kaplan–Meier survival analysis indicated significant differences in FAW survival days among the tested plants (Fig. 4). Notably, FAW fed on signal grass had a shorter survival duration (7 days) compared to corn plants (15 days), while FAW fed on the four tested Napier grass germplasms had survival durations ranging from 21 to 23 days with 50% survival probability. Furthermore, FAW larvae fed on signal grass and DIV-7 Napier grass exhibited lower pupation rates (8.8% and 45.9%) than those fed on corn plants, although no significant differences were observed for the other three Napier grass germplasms (G3, G4, and TS5) and corn plants (Table 2). No significant differences were noted in the adult emergence rate and adult ratio among all tested Napier grass plants, signal grass, and corn plants. Regarding FAW egg numbers, female moths preferred to lay more eggs on signal grass than on corn plants, while female moths laid less eggs on Napier grass germplasms than corn plants.

**Table 2:** Developmental parameters of FAW fed on Napier grass, signal grass, and corn. Significant differences between means are denoted by different letters (*p* < 0.05). Differences between Napier grass germplasm and corn are indicated by asterisks: * for *p* < 0.05, and *** for *p* < 0.001. Non-significant differences are denoted by “n.s.“. Error bars represent the standard error (S.E.)

Species	*Zea mays*		*Pennisetum purpureum*								*Brachiaria brizantha*	
			DIV-7		G4		G3		TS5			
Larval Weight ± S.E. (mg)	278.9 ± 10.9	a	72.4 ± 5.5	b	88.0 ± 6.6	b	100.2 ± 9.6	b	84.3 ± 13.1	b	57.7 ± 9.1	b
Larval Period ± S.E. (day)	13.4 ± 0.1	c	21.8 ± 0.6	a	19.6 ± 0.4	b	19.4 ± 0.5	b	20.2 ± 0.7	ab	22.5 ± 0.8	a
Pupation Rate (%)	71.1		45.9	*	59.0	n.s.	55.3	n.s.	54.5	n.s.	8.8	***
Pupal Weight ± S.E. (mg)	164.9 ± 4.6	a	119.2 ± 4.2	c	159.6 ± 3.9	a	148.9 ± 5.4	ab	153.7 ± 6.2	ab	125.8 ± 7.0	bc
Adult Emergence Rate (%)	71.9		76.5.	n.s.	56.5	n.s.	47.6	n.s.	44.4	n.s.	66.7	n.s.
Adult (F/M)	10/13		9/4	n.s.	4/9	n.s.	7/3	n.s.	3/5	n.s.	2/2	n.s.
Egg / Female	594.3		102.7	***	414.3	***	368.7	***	654.7	n.s.	778.0	***

### Evaluate desmodium for the push plant

**Fig. 5:**
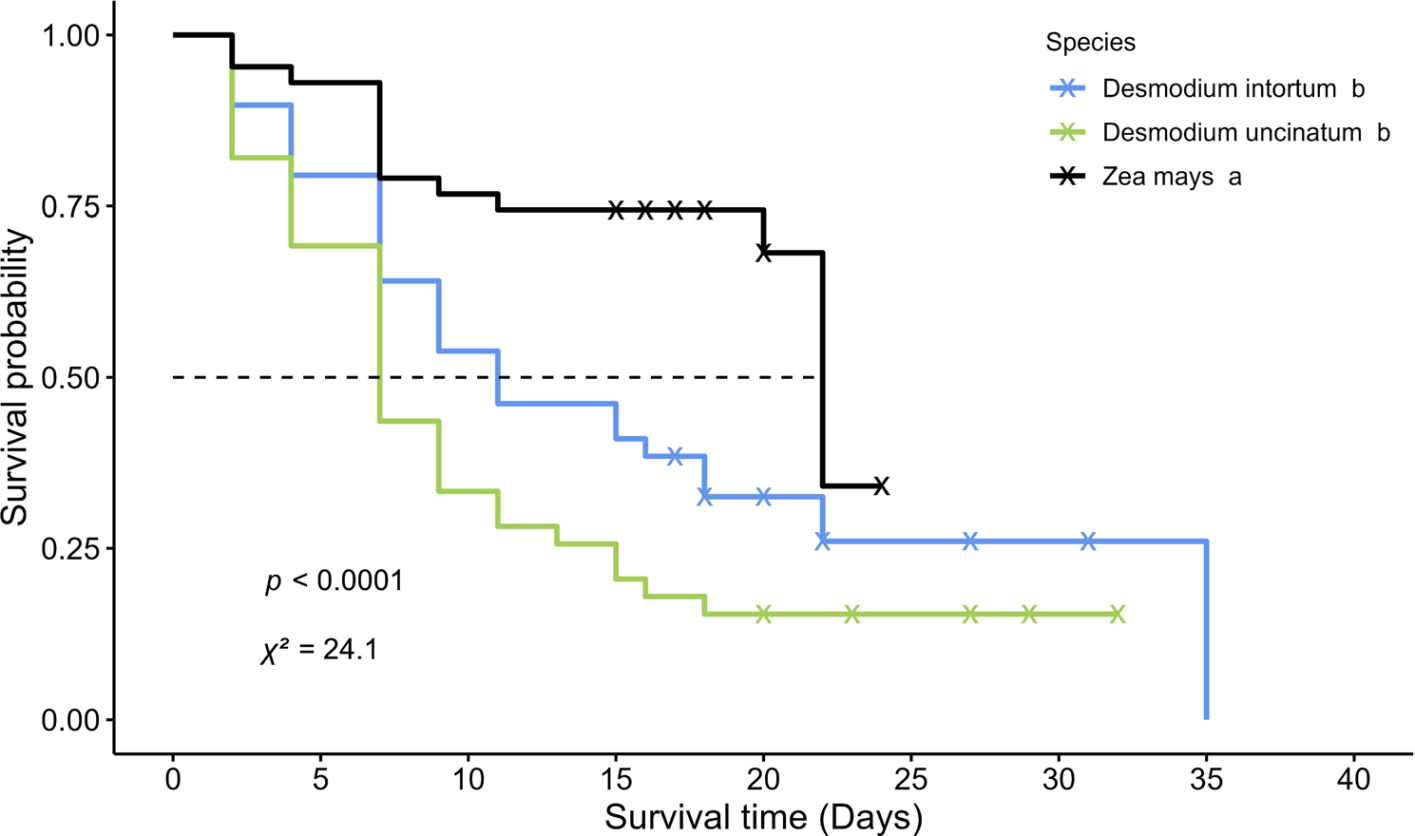
The survival probability of fall armyworm (FAW) larvae when fed on *Desmodium intortum*, *D. uncinatum* and corn. The survival curves were analyzed using the Kaplan-Meier log-rank test. Labels marked with the same letter indicate no significant difference. The symbol “x” denotes censored FAW larvae

Three *Desmodium* species (*D. tortuosum*, *D. intortum*, *D. uncinatum*) were first selected for assessing FAW performance. After evaluating their phenology, *D. tortuosum* was excluded due to the requirement for push plants to have prostrate traits. Following 11 feeding days, FAW larvae fed on *D. intortum* and *D. uncinatum* displayed lower larval weight than those fed on corn plants (Table 3), with *D. uncinatum* exhibiting the lowest larval weight. The larval period for FAW fed on both tested *Desmodium* species was longer than for those fed on corn plants (Table 3), and FAW fed on *D. uncinatum* had the longest larval developmental period. Kaplan–Meier survival analysis indicated significant differences in FAW survival days among *Desmodium* species (Fig. 5). Specifically, FAW fed on *D. intortum* and *D. uncinatum* had survival durations of 7 and 11 days, respectively, with 50% survival probability. In contrast, FAW larvae fed on corn plants had a 22-day survival duration with 50% survival probability. Additionally, FAW larvae fed on *D. intortum* and *D. uncinatum* exhibited lower pupation rates (28.2% and 15.4%) than those fed on corn plants (Table 3). No significant differences were observed in the adult emergence rate and adult ratio among tested *Desmodium* species and corn plants.

**Table 3:** Developmental parameters of FAW fed on *Desmodium intortum*, *D. uncinatum* and corn. Significant differences between means are denoted by different letters (*p* < 0.05). Differences between *Desmodium* species and corn are indicated by asterisks: *** for *p* < 0.001. Non-significant differences are denoted by “n.s.“. Error bars represent the standard error (S.E.)

Species	*Zea mays*		*Desmodium intortum*		*Desmodium uncinatum*	
Larval Weight ± S.E. (mg)	131.5 ± 9.1	a	76.7 ± 14.9	b	19.9 ± 5.1	c
Larval Period ± S.E. (day)	18.2 ± 0.3	c	21.1 ± 1.3	b	27.2 ± 2.0	a
Pupation Rate (%)	69.8		28.2	***	15.4	***
Adult Emergence Rate (%)	75.9		72.7	n.s.	50.0	n.s.
Adult (F/M)	10/12		4/4	n.s.	0/3	n.s.

## Discussion

In Taiwan, several lepidoptera species, such as *Ostrinia furnacalis* and *Helicoverpa armigera*, would cause severe damage on corn yield. Furthermore, the recent invasion of FAW further make a significant threat on the major agronomy crops. Therefore, it is an urgent need to find the suitable pest control methods on corn management besides spraying pesticides. The tested Napier grass germplasms exist highly antibiotic effect on FAW with low leaf consumption and high mortality rate. It indicated that they are not the optimum host plants for FAW. In addition, the results of FAW bioassay on Napier grass were further indicated that these potential trap plants would effective lower the number of FAW. However, it may question about the attractiveness of these trap plants on FAW female moths. In the field data, we showed that some of them (G3, G4, and DIV-7) would highly attract FAW female moths to oviposition based on the higher leaf damage scores. It implied these three Napier grass germplasms would emit more specific volatile compounds which highly attracted FAW female moths than FAW-preferred host plants, corn. Four volatiles (hexanal, (E)-2-hexenal, (Z)-3-hexen-1-ol, and (Z)-3-hexen-1-yl acetate) were higher in Napier grass (Chamberlain et al. 2006). Besides, we noticed that most of Napier grass have high density of trichome, not only on leaves but also on the stems, which would further contribute the antixenosis effect which did not show in our bioassay data.

*Desmodium* spp. serve various purposes, including use as green manure, host plants for butterflies, and cover crops. Species like *D. incanum* DC. var. *incanum*, *D. triflorum*, and *D. sequax* are favored by butterflies such as Long-tailed Skipper (*Urbanus proteus* domingo), Dorantes Longtails (*U. dorantes* santiago), and *Zizula hylax* hylax Fabricius as host plants (Fernández-Hernández 2007; Rayalu et al. 2012; Yang and Yang 2008). Conversely, *Pyrgus oileus* oileus, *Leptotes cassius* theonus, and *U. dorantes* Santiago have been observed visiting flowers of *D. incanum* var. incanum or *D. scorpiurus* (Sw.) var. *scorpiurus*. *D. sequax* Wall (Fernández-Hernández 2007) serves as a herbivorous plant for butterflies like the Sullied Brown Glider (*Neptis nata* lutatia) and the Pingshan Gray Butterfly (*Rapala nissa* hirayamana). *D. gangeticum* (L.) DC. exhibits robust growth and is utilized as ground cover to suppress weeds, with its stem fiber also finding application in paper-making (Lin 2005).

Despite being considered a weed in many regions due to its tenacity, *D. incanum* is valued for its ability to enhance soil fertility through nitrogen fixation, making it a popular choice for biological mulch and green manure. Additionally, it serves as a nutritious feed for various animals.

One notable application is the push-pull system, widely adopted by small farmers in Africa for corn or sorghum cultivation, which involves the use of *D. intortum* (DC.) and *D. uncinatum*. In our study, we observed that the Fall Armyworm (FAW) exhibited inferior performance on *D. intortum* and *D. uncinatum*. Furthermore, *Desmodium* spp. possess numerous non-glandular trichomes that impede the movement of FAW larvae and moths (Erdei et al., 2024). Consequently, *D. intortum* and *D. uncinatum* emerge as promising candidates for the push plant in the push-pull system in Taiwan.

*Desmodium* spp. play a crucial role in the push-pull farming system, providing not only insect control but also aiding in the management of parasitic weeds like Striga. Studies have shown that allelochemicals, such as isoschaftoside and isoflavanones, found in the root exudates of *D. uncinatum*, can influence the development of Striga (Hooper et al. 2010; Tsanuo et al. 2003). However, in Taiwan, parasitic weeds are less of a concern, as there are no reports of crop damage caused by the three *Sphenoclea* spp. found locally (*S. lutea*, *S. Masuria*, and *S. crispate*) (Wang et al. 2012). Additionally, these *Sphenoclea* spp. are considered endangered species in Taiwan (Wang et al. 2012), due to their limited population numbers, they have minimal impact on crops.

The push-pull farming system, successful in Africa, faces challenges in translation to Taiwan and other developed countries. *Desmodium*, despite its advantages as a push plant, grows relatively slowly compared to major crops, requiring time to establish populations before planting corn and sorghum. Therefore, this system may be more suitable for no-tillage farming, unlike the conventional system involving multiple tillages. Additionally, while *Desmodium*, Napier grass, and signal grass can serve as animal feed, small-scale farmers in Taiwan may not economically benefit from using these plants for feed. Enhancing the efficacy of this system in Taiwan remains a challenge. Economic benefits compared to synthetic pesticides or bio-pesticides will determine its widespread adoption and sustainability in Taiwan.

## Conclusions

This study is focused on selecting effective pull and push plants against FAW. Among 50 Napier grass germplasms, 35 displayed significant resistance to FAW, with 3 attracting more female moths for oviposition. Further evaluation of four Napier germplasms and signal grass revealed their efficacy in reducing FAW larval weight and survival duration. Among *Desmodium* species, *D. uncinatum* showed promising results in inhibiting FAW development. These findings highlight the potential of Napier grass and signal grass as pull plants and *D. uncinatum* as a push plant in FAW management strategies in Taiwan.

## Data Availability

The data used and analyzed for the current study can be obtained from the corresponding author.
